# Soil Microbial Community Response to Nitrogen Application on a Swamp Meadow in the Arid Region of Central Asia

**DOI:** 10.3389/fmicb.2021.797306

**Published:** 2022-01-20

**Authors:** Yang Hu, Mo Chen, Zailei Yang, Mengfei Cong, Xinping Zhu, Hongtao Jia

**Affiliations:** ^1^College of Resources and Environment, Xinjiang Agricultural University, Urumqi, China; ^2^College of Grassland Science, Xinjiang Agricultural University, Urumqi, China; ^3^Xinjiang Key Laboratory of Soil and Plant Ecological Processes, Urumqi, China

**Keywords:** nitrogen application, swamp meadow, microbial community, bacteria, fungi

## Abstract

Although a large number of studies have reported the importance of microbial communities in terrestrial ecosystems and their response to nitrogen (N) application, it is not clear in arid alpine wetlands, and the mechanisms involved need to be clarified. Therefore, the response of the soil microbial community in a swamp meadow to short-term (1 year) N application (CK: 0, N1: 8, N2: 16 kg⋅N⋅hm^–2^⋅a^–1^) was studied using 16S/ITS rRNA gene high-throughput sequencing technology. Results showed that N application had no significant effect on soil microbial community diversity, but significantly changed soil bacterial community structure. N1 and N2 treatments significantly reduced the relative abundance of Chloroflexi (18.11 and 32.99% lower than CK, respectively). N2 treatment significantly reduced the relative abundance of Nitrospirae (24.94% lower than CK). Meanwhile, N application reduced the potential function of partial nitrogen (N) and sulfur (S) cycling in bacterial community. For example, compared with CK, nitrate respiration and nitrogen respiration decreased by 35.78–69.06%, and dark sulfide oxidation decreased by 76.36–94.29%. N application had little effect on fungal community structure and function. In general, short-term N application directly affected bacterial community structure and indirectly affected bacterial community structure and function through available potassium, while soil organic carbon was an important factor affecting fungal community structure and function.

## Introduction

Due to the rapid development of industry and agriculture, the amount of N-containing compounds entering the soil through atmospheric deposition has increased dramatically (Shi et al., 2016; Zhou et al., 2017), and the deposition rate of atmospheric N caused by human activities has increased by 3–5 times (Galloway et al., 2004). China has become the third-largest N sink in the world (Zhu et al., 2019). N deposition in the soil breaks the original N cycle and affects the structure and function of the ecosystem (Peter, 2007; Vitousek et al., 2010). Excessive N input will lead to soil acidification (Raza et al., 2020), reduction of plant diversity (Liu et al., 2020a), and changes in soil microbial community composition (Shi et al., 2016; Zhou et al., 2017).

The soil microbial community is an important biotic component of soil and an important driving force of many soil ecological processes (Wang et al., 2021). Due to the high sensitivity of soil microbes, the increase in N deposition is bound to have a significant impact on the soil microbial community; however, the response to N deposition often differs in different ecosystems, and the relationship between the soil microbial community and soil factors is uncertain (Xiao et al., 2020; He et al., 2021). Therefore, monitoring the response of soil microorganisms to N application is helpful to evaluate the changes of ecosystem processes driven by soil microbiota.

At present, a large number of studies have found various responses of soil microbial community to N application (Wang C. et al., 2018; Wang H. et al., 2018). N application significantly altered the soil bacterial community structure of semi-arid and sub-humid grassland, with significant changes found in seven phyla of bacteria in 0–10 cm layers (Zeng et al., 2016). Three-year N application would significantly increase the fungi α-diversity in semi-arid grassland (Zhang et al., 2018). But another study on a semi-arid grassland found that 2-year N application did not affect its soil microbial composition and diversity (McHugh et al., 2017). In addition, 20-year N application had no significant effect on the total amount and activity of the bacterial community in forest topsoil, nor on the composition, diversity, and richness of the fungal community (Freedman et al., 2015). As a result, the results vary depending on the ecosystem. N application has a significant effect on the functional metabolism of the soil microbial community. N application significantly affected the metabolic functional potential of bacterial and fungal communities under the forest soil (Li J. et al., 2021). N application also promoted many metabolic processes of the microbial community, especially carbohydrate and amino acid related metabolic processes and archaea mediated ammonia oxidation, but N application also reduced N_2_ fixation and signal transduction (Li B. et al., 2021).

Moreover, many studies focus on forest and grassland ecosystems, but few on the alpine wetland ecosystem. Changes in the soil microbial community structure are related to several environmental factors, such as soil pH (Zeng et al., 2016), nutrient availability (Liu et al., 2020a), soil organic carbon (SOC) (Wang H. et al., 2018), and vegetation types (Li et al., 2020). At present, most studies consider soil pH as the main driving force for the change in the soil microbial community (Yuan et al., 2016; Wu et al., 2019). The response of soil microorganisms to N application also depends on the background level of atmospheric N deposition. With an increase in N application, the fungal diversity increased in soil with low atmospheric N deposition, whereas it decreased in soil with high atmospheric N deposition (Moore et al., 2020). Therefore, the factors driving change in the soil microbial community vary in different ecosystems. Moreover, research on the characteristics of the soil microbial community in the alpine wetland ecosystem is important, and the factors causing soil microbial community change under N application are not clear.

The alpine wetland is a unique ecosystem formed by land–water interaction. Compared with other wetland ecosystems, the alpine wetland ecosystem is more vulnerable and sensitive to atmospheric changes such as N deposition (Cao et al., 2017). Bayinbuluk alpine wetland is located at the south foot of Tianshan Mountain. Tianshan Mountain is the farthest from the ocean in the world and the largest mountain system in the world’s arid area. Bayinbuluk alpine wetland is a typical and important wetland in the arid area of Central Asia. It is a world natural heritage site and plays an important role in water regulation, water storage, and maintaining regional water balance (Shao and Gao, 2018; LiuSui et al., 2019). With a rapid increase in the N deposition rate, N is bound to affect the structure and function of the soil microbial community, affecting the ecosystem nutrient cycle. Therefore, using 16S/ITS rRNA gene high-throughput sequencing technology to study the effects of different N concentrations on diversity, structure, and function of the soil microbial community, block design experiments were performed in the Bayinbuluk alpine swamp meadow. The objectives of this study were (1) to explore the effects of N application on the diversity, structure, and function of soil microbial community in swamp meadow and (2) to reveal the driving factors of soil microbial community change in swamp meadow under the condition of N application.

## Materials and Methods

### Overview of the Study Area

**FIGURE 1:**
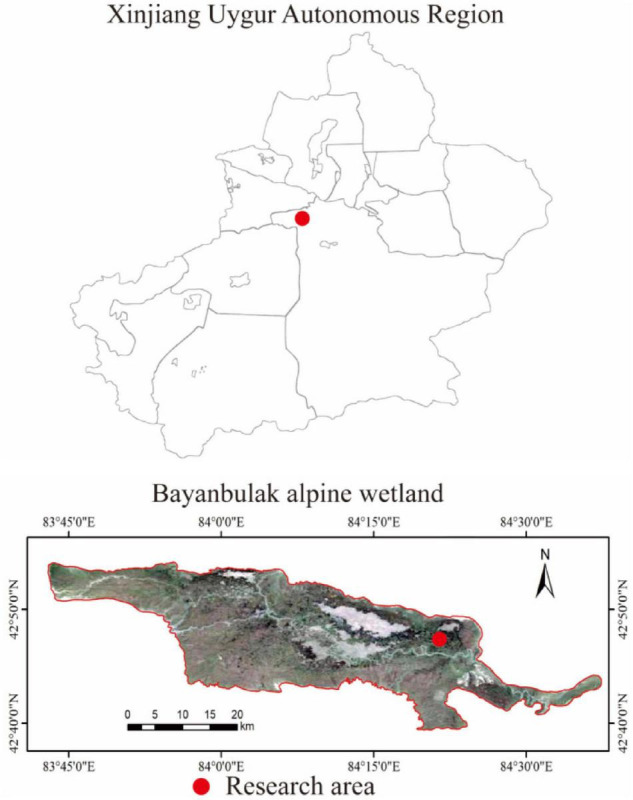
Geographical location of the study area.

This study was conducted in the Bayinbuluk alpine wetland, Hejing County, Xinjiang Uygur Autonomous Region, China (82°59′–84°35′E, 42°40′–43°00′N) (Figure 1). The study area is about 770 km^2^ and at an altitude of 2,300–3,042 m, belonging to the temperate continental arid climate. The annual average temperature is −4.6°C, and the extreme maximum and minimum temperatures are 28 and −48.1°C, respectively. The average annual precipitation is 273 mm, annual evaporation is 1,250 mm, and the average annual relative humidity is 60%. The swamp meadow soil water content is between 50 and 70%, and the groundwater level is about 120 cm. The dominant vegetation is *Carex melanantha* and *Triglochin maritimum* (LiuSui et al., 2019; Chen et al., 2021).

### Experimental Design

**FIGURE 2:**
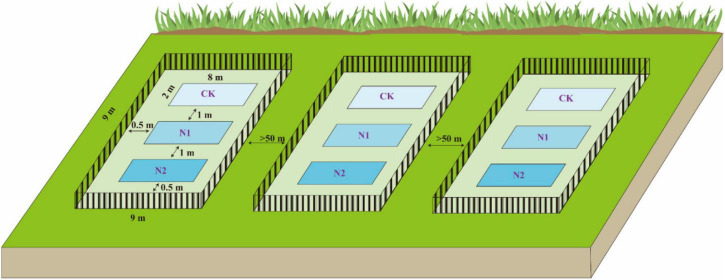
Layout diagram of field block design experiments.

At the end of the plant growing season in October 2018, using the block design method, three blocks (9 m × 9 m) were randomly established in the Bayinbuluk alpine swamp meadow (Figure 2). The distance between each block is more than 50 m, and a 1-m high steel fence was used to prevent human and animal interference. In the beginning, each block was set up with three plots (2 m × 8 m) according to three N application treatments, and there was a buffer of 1 m in the middle of plots to prevent the interaction between different treatments. According to the N deposition (8 kg⋅N⋅hm^–2^⋅a^–1^) of the Bayinbuluk in 2011 (Li et al., 2012), the N application concentrations in this study were set at CK (0 kg⋅N⋅hm^–2^⋅a^–1^), N1 (8 kg⋅N⋅hm^–2^⋅a^–1^), and N2 (16 kg⋅N⋅hm^–2^⋅a^–1^), respectively, and three replicates of each treatment. The required amount of urea was weighed according to the different N application concentrations and fully dissolved in distilled water, sprayed evenly on different treatments in each plot. The same amount of distilled water was sprayed in the control to eliminate the influence of soil water. In October 2019, soil samples at 0–10 cm depth in each plot were collected by the five-point sampling method. One soil sample was dried in the shade and screened to determine soil moisture content, pH, soil organic carbon (SOC), available nitrogen (N), available phosphorus (P), available potassium (K), total carbon (C), and total nitrogen (N). Another soil sample was stored in a cold storage tube in a liquid N tank (−20°C) for high-throughput sequencing analysis of the soil microbial community structure.

### Determination of Soil Basic Indices

Soil pH was measured at a water/soil ratio of 2.5:1 using a pH meter (Mettler Toledo FE28-Standard, Switzerland). The total SOC content was determined by the H_2_SO_4_–K_2_Cr_2_O_7_ external heating method. Available N was determined by the alkali diffusion method. Soil available P was determined by spectrophotometry (Shimadzu UV-1780, Japan) (Shao et al., 2019). Available K was determined by flame photometry (Chen et al., 2021). Soil moisture content was measured by the drying method. Soil total C and total N was determined by an elemental analyzer (Euro EA3000, Italy).

### DNA Extraction and Sequencing

Bacterial and fungal DNA was extracted using the PowerSoil DNA Isolation Kit (MoBio, United States). Common primers were used to amplify the 338F (5′-ACTCCTAGGGAGCA-3′) and 806R (5′-GGACTCHVGGGTWTTAT-3′) binding adaptors and barcode sequences in the V3–V4 region of the bacterial 16S rRNA gene (Christian et al., 2013). Fungal ITS1 primers were used for ITS1 (5′-CTGTCATTAGGGAGAGAGA-3′), and ITS2 (5′-GCTGCGTTCTTCATCGATGA-3′) was amplified by combining an adaptor sequence and a barcode sequence (Kõljalg et al., 2013). The resulting products were purified, quantified, and homogenized to form a sequencing library. The constructed library was first inspected, and the qualified library was sequenced with Illumina HiSeq 2500 (Illumina, United States). All raw sequences have been deposited into a NCBI Sequence Read Archive with the BioProject accession: PRJNA771427.

### Bioinformatics Analysis

Overlapping reads of the samples were spliced using FLASH software (version 1.2.11^1^), and the obtained spliced sequences were the original tag data. Trimmomatic software (version 0.33) was used to filter the raw tags obtained by splicing to obtain high-quality tag data. UCHIME software (version 8.1) was used to identify and remove chimeric sequences to obtain the final data. USEARCH software (version 10.0) was used to cluster tags and obtain operational taxonomic units (OTUs) with 97% similarity. Tags were based on bacterial 16S sequences from the SILVA database (Release132^2^) and fungal ITS sequences from the UNITE database (Release 8.0^3^). RDP Classifier (version 2.2^4^) was used to classify taxa with a minimum reliability estimate of 80%. Alpha diversity of the microbial community was analyzed by Mothur (version v.1.30^5^) using the Chao1 and ACE richness estimators, and the Shannon–Wiener and Simpson diversity indices were used to measure microbial diversity. The FAPROTAX database^6^ connecting species classification and function annotation was constructed, and then the OTU classification table and FAPROTAX database were contacted to predict the function of the bacterial community (Louca et al., 2016). Fungal gene sequence information was obtained from FUNGuild,^7^ which is associated with fungal function, and was used to predict fungal community function (Xie et al., 2020). Species relative abundance in different samples was determined using STAMP^8^ with *G*-test and Fisher inspection methods. The two sample *t*-test was used to determine significant differences with a *P*-value threshold of 0.05 (Chen et al., 2021).

### Data Analysis

R (version 4.0.2) was used to analyze and plot the data, and analysis of variance and least significant difference test of the agricolae software package (version 1.3.3) was used to test significant differences. Ggalluvial software package (version 0.11.3) was used to draw the superimposed column diagram of microbial community relative abundance. We defined the relative abundance of top 10 phylum level microorganism as dominant phylum. Linear discriminant analysis (LDA) effect size (LEfSe) analysis^9^ was used to estimate the relative abundance of species, with a logarithmic LDA score threshold of 3.0 (Zhou et al., 2019). LEfSe analysis was performed to identify significant differences from phylum to genus level among the three treatment groups and to determine the most likely characteristics to explain the differences between the categories (Segata et al., 2011). A principal coordinate analysis (PCoA) based on bray-Curtis distance was constructed using a vegan software package (version 2.5.6) to measure microbial community β-diversity, and pairwise differences were further compared based on Wilcoxon rank-sum test. The vegan software package (version 2.5.6) was used to rank microbial and soil properties by redundancy analysis (RDA) (Leps and Smilauer, 2003). The Monte Carlo permutation test was used to analyze the significance of soil properties (permu = 999). The structural equation modeling (SEM) analysis was performed using SPSS Amos 24 (IBM, United States). Analysis of microbial metabolic function was performed by STAMP and plotted with Origin Pro 8. SEM was used to understand how soil characteristics mediate changes in the soil microbial community structure and function under the condition of N application. After all OTU of soil bacteria and fungi were ranked by principal component analysis (PCA), the first principal component (PC1) was used in subsequent SEM analysis (Liu and Zhang, 2019).

## Results

### Effect of N Application on Soil Chemical Properties

N application did not significantly affect soil moisture, total C, total N, soil pH, and available P content (*P*> 0.05) (Table 1). Compared with CK, the SOC content after N1 treatment decreased by 4.19%. N1 and N2 treatments significantly increased soil available N content by 18.32 and 21.20%, respectively (*P* < 0.05). N2 treatment significantly increased the soil available K content by 12.50% (*P* < 0.05).

**TABLE 1:** Effects of N application on soil chemical properties.

Treatment	Moisture (%)	Total C (g⋅kg^–1^)	Total N (g⋅kg^–1^)	pH	SOC (g⋅kg^–1^)	Available N (mg⋅kg^–1^)	Available P (mg⋅kg^–1^)	Available K (mg⋅kg^–1^)
CK	54.33 ± 0.58a	86.67 ± 7.14a	7.1 ± 0.83a	8.07 ± 0.08a	70.58 ± 0.30a	78.02 ± 1.96b	77.41 ± 3.69a	326.67 ± 7.64b
N1	54.03 ± 0.45a	89.54 ± 1.53a	7.52 ± 0.91a	8.02 ± 0.01a	67.62 ± 0.20b	92.31 ± 0.43a	79.63 ± 3.72a	330.00 ± 5.00b
N2	54.10 ± 0.85a	85.78 ± 4.81a	7.28 ± 0.01a	8.03 ± 0.05a	72.56 ± 1.22a	94.56 ± 0.20a	78.56 ± 0.74a	367.50 ± 3.54a

*The same lowercase letter indicates no significant difference, but the difference is significant at the 0.05 level.*

### Effects of N Application on Soil Microbial Community Diversity and Structure

**FIGURE 3:**
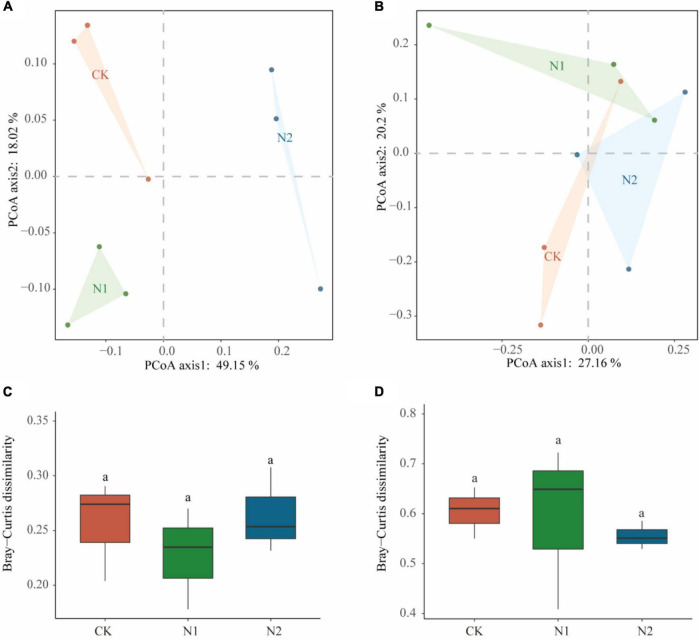
Effects of N application on soil microbial community β-diversity. **(A)** Bacterial community PCoA based on Bray-Curtis dissimilarity. **(B)** Fungal community PCoA based on Bray-Curtis dissimilarity. **(C)** Bacterial community β-diversity differences. **(D)** Fungal community β-diversity differences.

Sequencing all samples, we found that the number of bacterial OTUs was 1,284–1,401 and the number of fungal OTUs was 342–404 (Table 2). All OTUs were used for subsequent statistical analysis. In this study, N application did not significantly affect the number of OTUs and α-diversity indices (i.e., ACE, Chao1, Simpson, and Shannon–Wiener) of the soil microbial community (*P* > 0.05). PCoA analysis based on the Bray-Curtis distance matrix showed that under the condition of N application, the bacterial community was more dispersed than the fungal community (Figures 3A,B). N application on bacterial and fungal communities β-diversity had no significant effect (*P* > 0.05) (Figures 3C,D).

**TABLE 2:** Effects of N application on OTU number and diversity index of soil microorganisms.

Microbial species	Treatment	OTU	ACE index	Chao1 index	Simpson index	Shannon index
Bacteria	CK	1363.67 ± 32.7a	1572.94 ± 15.19a	1600.89 ± 16.91a	0.0058 ± 0.0009a	5.92 ± 0.10a
	N1	1373.33 ± 90.5a	1554.49 ± 122.38a	1578.09 ± 151.89a	0.0055 ± 0.0011a	5.99 ± 0.14a
	N2	1410.00 ± 54.4a	1503.50 ± 54.53a	1546.10 ± 72.43a	0.0057 ± 0.0018a	6.11 ± 0.13a
Fungi	CK	346.67 ± 2.08a	439.26 ± 48.44a	429.75 ± 20.30a	0.1116 ± 0.1077a	3.35 ± 0.7a
	N1	368.33 ± 4.04a	499.30 ± 93.94a	469.48 ± 41.58a	0.1244 ± 0.0491a	3.16 ± 0.30a
	N2	373.00 ± 31.00a	454.61 ± 29.03a	471.97 ± 32.06a	0.1365 ± 0.0476a	2.90 ± 0.28a

*The same lowercase letter indicates no significant difference, but the difference is significant at the 0.05 level.*

A total of 28 phyla were detected, and the dominant phyla (10 with the highest relative abundance) were selected to evaluate changes in soil bacterial community composition (Figure 9A). The relative abundance of Proteobacteria was the highest in all samples, and its average relative abundance was 31.52, 31.00, and 31.02% for CK, N1, and N2 treatments, respectively, followed by Acidobacteria (13.33, 15.20, 15.38%), Actinobacteria (12.23, 12.64, 11.76%), Chloroflexi (15.50, 12.68, 10.38%), Gemmatimonadetes (10.95, 12.38, 12.31%), and Bacteroidetes (8.97, 9.21, 10.80%). The relative abundance of Firmicutes, Verrucomicrobia, and Planctomycetes was also low. Among them, the relative abundance of Chloroflexi decreased significantly with the increase of N application concentration (*P* < 0.05). Compared with CK, N1 and N2 significantly decreased by 18.11 and 32.99%, respectively, while N2 significantly decreased the relative abundance of Nitrospirae by 24.94% (*P* < 0.05). A total of 8 fungal phyla were detected (Figure 9B), of which most were unclassified, the relative abundance for 66.51–81.04%. The relative abundance of Ascomycetes was the highest (24.64, 17.88, 26.26%). The relative abundance of other phyla, including Mortierellomycata, Glomeromycota, and Basidiomycota, was low.

**FIGURE 4:**
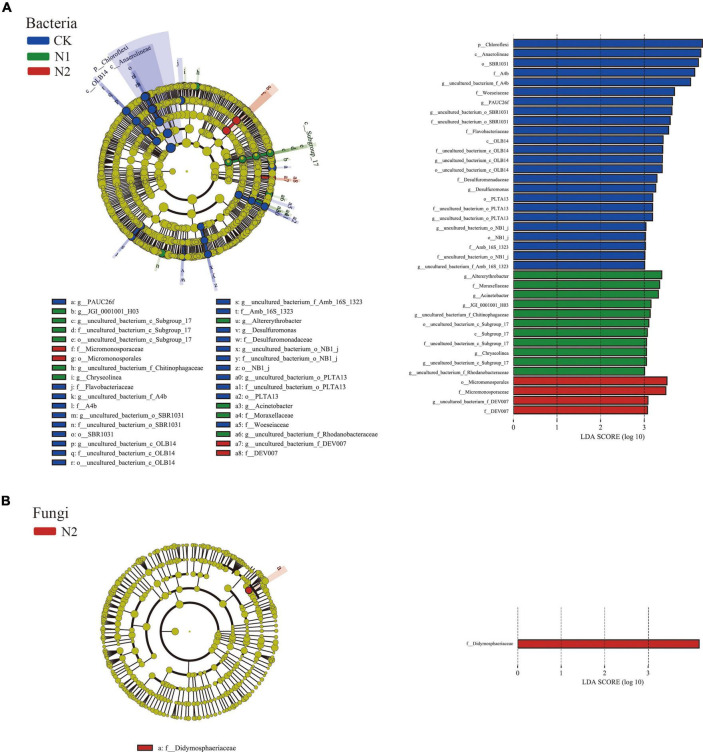
LEfSe analysis of soil microbial community structure after N application (phylum–genus level). **(A)** Bacterial LEfSe (LDA = 3.0). **(B)** Fungal LEfSe (LDA = 3.0).

LEfSe showed that 39 bacterial communities and 1 fungal community were significantly different; there were 24 biomarkers in CK, 11 biomarkers in N1, and 4 biomarkers in N2 (Figure 4A). The CK treatment group mainly included Chloroflexi (phylum), Anaerolineae (class), SBR1031 (order), A4b (family), and uncultured_bacterium_f_A4b (genus), among which the most abundant was Chloroflexi (phylum). In fungi, the relative abundance of unclassified fungi was high (66.51–81.04%) (Figure 4B), and there was no significant difference after N treatment. Only one fungal biomarker from Didymosphaeriaceae (family) was found after N2 treatment.

### Effects of N Application on Bacterial Functional Metabolism and Fungal Nutritional Patterns

**FIGURE 5:**
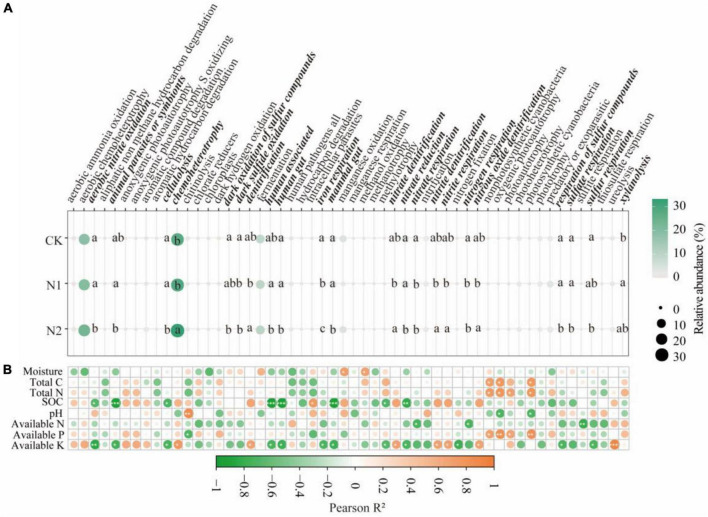
Prediction of soil bacterial function after N application and its correlation with soil factors. **(A)** Prediction of soil bacterial function. The same lowercase letter indicates no significant difference, but the difference is significant at the 0.05 level. **(B)** Correlation between bacterial function and soil factors. *P < 0.05, **P < 0.01, ***P < 0.001. Bacteria function with significant differences are highlighted in bold italics.

Through FAPROTAX functional annotation, bacterial communities showed 54 functions (Figure 5A). It contains many functions related to N and S cycling. The relative abundance of the 54 functions chemoheterotrophy (27.94–33.24%), aerobic chemoheterotrophy (18.55–23.27%), and fermentation (9.13–10.13%) were higher. Through divergence analysis, it was found that 22 of the 54 functions showed significant differences under N application treatment (*P* < 0.05). It is worth noting that most of the 22 different functions are related to the N and S cycling. In the N metabolism, N application significantly reduced nitrate respiration and nitrogen respiration (compared with CK, it decreased by 35.78–69.06%) (*P* < 0.05). N2 treatment significantly reduces aerobic nitrite oxidation and nitrate reductio (compared with CK, it decreased by 42.27 and 23.55%, respectively) (*P* < 0.05). The denitrification, nitrate denitrification, nitrite denitrification, nitrite respiration, and nitrous oxide denitrification of N2 treatment were significantly higher than those of N1 treatment (*P* < 0.05). In the S cycle, N application significantly reduced dark sulfide oxidation (compared with CK, it decreased by 76.36–94.29%) (*P* < 0.05). N2 treatment significantly reduced dark oxidation of sulfur compounds, respiration of sulfur compounds, sulfate respiration, and sulfur respiration (compared with CK, it decreased 89.18, 91.31, 87.30, and 92.37%, respectively) (*P* < 0.05). From the correlation analysis (Figure 5B), it can be seen that soil available K is significantly correlated with most bacterial community functions, followed by SOC (*P* < 0.05).

**FIGURE 6:**
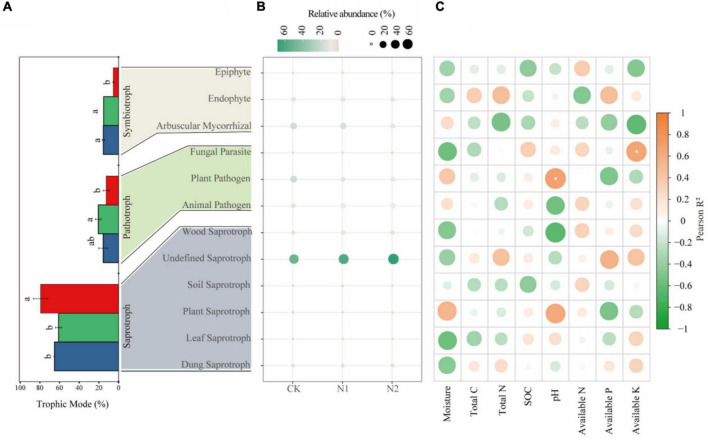
Changes in soil fungi according to main trophic modes and guild categories (FUNGuild) after N application. **(A)** Changes in fungi according to trophic mode. The same lowercase letter indicates no significant difference, but the difference is significant at the 0.05 level. **(B)** Main fungal guilds. Colors indicate different fungal types, and circle sizes indicate their relative abundance. **(C)** Correlation between fungal function and soil factors. *P < 0.05.

Saprotrophs dominated the fungal community, accounting for 54.92–66.06% in the three treatments (Figure 6A). The proportion of pathotrophs, saprotrophs, and symbiotrophs corresponded to 15.65, 65.18, and 15.48% after CK treatment; 20.61, 61.01, and 15.20% after N1 treatment; and 59, 79.23, and 5.34% after N2 treatment. The results showed that N application significantly affected the main trophic patterns of fungi. Compared with CK, N2 treatment significantly increased the proportion of saprotrophs by 21.56% and significantly reduced the proportion of symbiotrophs by 65.50% (*P* < 0.05). Based on the FUNGuild trophic modes (Figure 6B), the main fungal guilds in the swamp meadow soil were undefined saprotroph (48.98–73.82%), plant pathogen (5.19–19.58%), arbuscular mycorrhizal fungi (4.31–18.93%), animal pathogen (4.45–9.30%), endophyte (4.94–5.09%), and wood saprotroph (2.06–4.92%). The rest accounted for a small proportion (<1%), such as plant saprotroph, soil saprotroph, dung saprotroph, epiphyte, and mycoparasite. N application had no significant effect on the main fungal guilds (*P* > 0.05). According to the correlation analysis (Figure 6C), the correlation between soil factors and main fungal guilds is small; only AK and Fungal Parasite, and pH and Plant Parasite are significantly correlated (*P* < 0.05).

### Relationship Between Microbial Community Structure and Soil Properties

**FIGURE 7:**
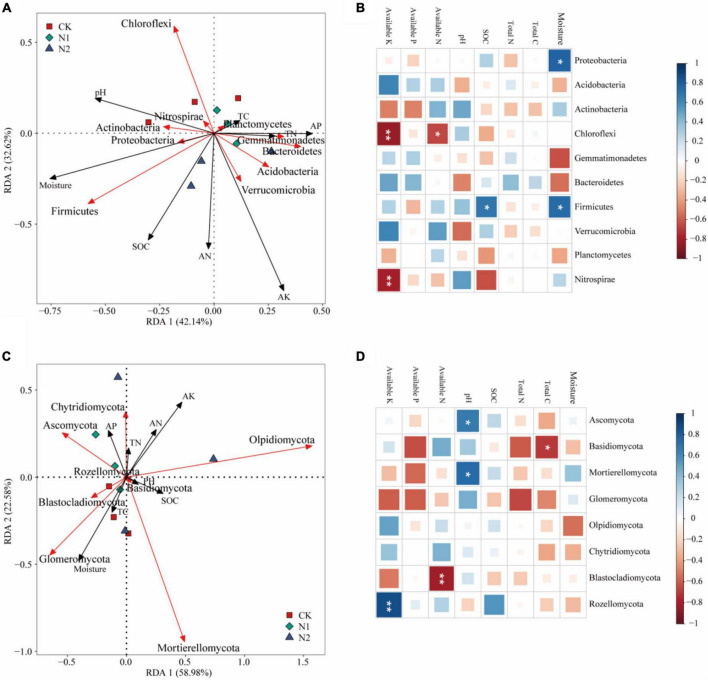
Effects of soil chemical properties on structure and function of soil microbial community after N application. **(A)** Redundancy analysis of bacterial community structure and soil properties. Red arrows indicate soil bacteria, and black arrows indicate soil chemical properties (length = level). **(B)** Correlation between bacterial community (phylum) and soil chemical properties. *P < 0.05, **P < 0.01. **(C)** Redundancy analysis of fungal community structure and soil properties. Red arrows indicate soil fungi, and black arrows indicate soil chemical properties (length = level). **(D)** Correlation between fungal community (phylum) and soil chemical properties. *P < 0.05, **P < 0.01.

The effect of soil factors on the microbiota level was determined by RDA (Figure 7). Overall, the two dimensions (RDA1 and RDA2) explained 74.76% of the changes of soil bacterial community (Figure 7A). According to RDA and correlation analysis (Figures 7A,B), soil available K has a significant negative correlation with Chloroflexi and Nitrospirae, available N also has a significant negative correlation with Chloroflexi, SOC and moisture have a significant positive correlation with Firmicutes, and moisture has a significant positive correlation with Proteobacteria (*P* < 0.05). The two dimensions (RDA1 and RDA2) explained 81.56% of the change of soil fungal community (Figure 7C). According to RDA and correlation analysis (Figures 7C,D), available K was significantly positively correlated with Rozellomycota, available N was significantly negatively correlated with Blastocladiomycota, and pH was significantly positively correlated with Ascomycota and Mortierellomycota. Total C was significantly negatively correlated with Basidiomycota (*P* < 0.05). Monte Carlo permutation test showed that the soil bacterial community structure was controlled by soil available K and moisture (*P* < 0.01), while the soil fungal community structure was not significantly affected by soil factors (Table 3) (*P* > 0.05).

**TABLE 3:** Monte Carlo permutation test of soil factors on microbial community structure.

Indication	Bacteria				Fungi			
	RDA1	RDA2	r^2^	*P*	RDA1	RDA2	r^2^	*P*
Moisture	–0.949	–0.315	0.634	**0.041**	–0.646	–0.763	0.394	0.214
TC	0.863	0.504	0.019	0.943	–0.519	–0.855	0.056	0.864
TN	0.998	–0.056	0.080	0.804	0.109	0.994	0.028	0.864
pH	–0.458	–0.889	0.437	0.181	0.957	–0.289	0.106	0.753
SOC	–0.944	0.331	0.337	0.278	0.924	–0.381	0.012	0.967
AN	–0.041	–0.999	0.405	0.223	0.678	0.735	0.139	0.680
AP	0.999	–0.005	0.205	0.502	–0.498	0.868	0.096	0.745
AK	0.348	–0.938	0.854	**0.005**	0.737	0.675	0.407	0.188

*RDA1 indicates the cosine value of the angle between soil factor and ordination axis, and RDA2 indicates the correlation between soil factor and ordination axis. r^2^ is the determination coefficient of soil factors in species distribution. The smaller r^2^, the smaller the influence of soil factors on species distribution. P is the significance test of correlation. Bold values indicates P < 0.05.*

**FIGURE 8:**
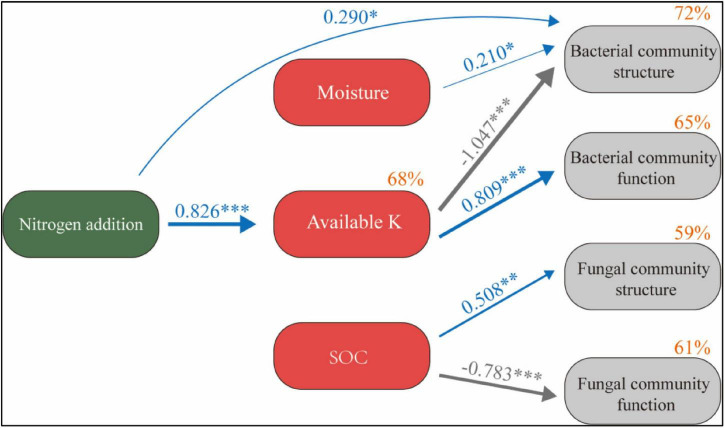
Structural equation model of N application on the soil microbial system reveals the relationship between SOC, moisture, available K, and microbial community structure and function. Width of the arrow head indicates the strength of the relationship. Blue arrows indicate a significant positive relationship, and gray arrows indicate a significant negative relationship (*P* < 0.05). Non-significant paths have been deleted. Percentages close to variables refer to the variance accounted for by the model (R^2^). *P < 0.05, **P < 0.01, ***P < 0.001.

SEM describes the direct and indirect effects of N application and soil factors on microbial communities (χ^2^ = 11.81, *P* = 0.298) (Figure 8). The model explained 68% of the changes in available K, 72% of the changes in bacterial community structure, 65% of the changes in bacterial community function, 59% of the changes in fungal community structure, and 61% of the changes in fungal community function. N application had no significant direct effect on soil SOC and moisture (*P* > 0.05). It has a significant positive effect on available K and has a significant negative effect on bacterial community structure and a significant positive effect on bacterial community function through available K (*P* < 0.05). Soil moisture also had a significant positive effect on bacterial community structure (*P* < 0.05). SOC had a significant positive effect on fungal community structure and a significant negative effect on fungal community function (*P* < 0.05).

## Discussion

N application will have a profound impact on soil nutrient cycling mediated by the soil microbial community. Therefore, it is of great significance to comprehensively study the changes and influencing factors of soil microbial community structure and function under the condition of N application (Allison and Martiny, 2008; Zhu et al., 2020). Overall, in this study, short-term N application had no significant effect on soil microbial community diversity, but directly affected the bacterial community structure and indirectly affected the bacterial community structure and function through available K. SOC is an important factor affecting the structure and function of fungal community.

In this study, short-term N application to swamp meadow soil did not significantly affect soil microbial community α-diversity and β-diversity (*P* > 0.05) (Figure 3). This is consistent with the previous studies on forest soil and grassland (Freedman et al., 2015; McHugh et al., 2017). But N application will also reduce microbial diversity (Wang C. et al., 2018). This is different from the results of this study. First, this may be because the alpine wetland in this study is different from other ecosystems in that soil microorganisms have different sensitivity to exogenous N input (Ramirez et al., 2010). In addition, 2 years of N application had no significant effect on soil microbial community diversity (McHugh et al., 2017). But 10 years of N application reduced soil bacterial diversity in subtropical forests (Wu et al., 2019). Therefore, in this study, N application had no significant effect on soil microbial community diversity may be due to the short-time of nitrogen application. Second, when N application level was low (less than 160 kg⋅N⋅hm^–2^⋅a^–1^), N application had no significant effect on soil bacterial diversity in semi-arid grassland (Liu et al., 2020a). In this study, the N application level was also within this range, so a small amount of N application had no significant effect on the diversity of the soil microbial community. This may be attributed to the greater plasticity, higher population density, and greater transmission capacity of microbial communities (Shade et al., 2012; Liu et al., 2020a). Third, in this study, N application has no significant effect on soil pH, which can also be used to explain why there is no significant difference in microbial community diversity (Table 1). A large number of studies showed that soil pH is the most important factor to predict the change of microbial community diversity. Some studies have found that if the soil pH value increases, the α-diversity of the microbial community increased; if the soil pH value is reduced, the α-diversity was be reduced; if there is no effect on soil pH, the α-diversity was not be changed (Zhou et al., 2020).

**FIGURE 9:**
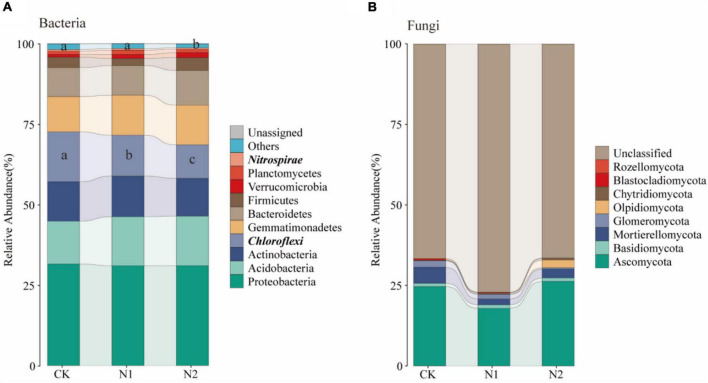
Changes in soil microbial community structure after N application. **(A)** Changes in the top 10 bacteria at the phylum level. **(B)** Changes in fungi at the phylum level. Phylum level with significant differences are highlighted in bold italics.

In this study, Proteobacteria, Acidobacteria, Actinobacteria, Chloroflexi, Gemmatimonadetes, and Bacteroidetes were the main bacterial communities under the swamp meadow soil (Figure 9A), which is consistent with that found in other wetland ecosystems (Xiong et al., 2014; Li et al., 2016; Xie et al., 2020). It can participate in energy metabolism and plays a key role in nutrient cycling and the ecological environment (Huhe et al., 2017; Eichorst et al., 2018; Fang et al., 2018). It is worth noting that the relative abundance of Gemmatimonadetes in the present study reached 10.95–12.38%, which is higher than that reported in previous studies (Gu et al., 2018; Shao et al., 2019; Zhou et al., 2019; Wang et al., 2020). Gemmatimonadetes are widely distributed in various ecosystems. Although Gemmatimonadetes are not dominant in nature, they still play a key ecological function because of their metabolic and physiological diversity; their relative abundance is negatively correlated with soil moisture and they grow in dry soils (DeBruyn et al., 2014). This study was conducted in the alpine wetland in the arid region of Central Asia. When samples were collected, the soil moisture content was about 54.15%, lower than that of other wetland ecosystems, thereby improving the survival of Gemmatimonadetes.

The change of soil microbial community composition is usually related to the change of ecosystem environment, such as N application. Most studies found that N application has significant impact on soil microbial community structure (Shi et al., 2016; Zhou et al., 2017). LEfSe analysis also confirmed this point. N application significantly changed the soil bacterial community structure, but had little effect on the fungal community structure (Figure 4). On the one hand, this may be due to their different life strategies (Sessitsch et al., 2001). On the other hand, this is due to the difference in the number of bacteria and fungi, which is also confirmed by the difference in the number of OTU of bacteria and fungi in this study (Table 2). Previous studies have shown that bacteria are more susceptible to environmental influences than fungi (de Vries et al., 2018; Yang and Wu, 2020).

N application reduced the relative abundance of soil Actinobacteria and Nitrospirae (Wang C. et al., 2018). The change of Nitrospirae in this study is consistent with this. At the same time, Chloroflex, Anaerolineae, SBR1031, and A4b also decreased significantly with the N application, which is consistent with previous studies (Zeng et al., 2016; Liu et al., 2020a). These changes can be explained by the co-trophic hypothesis, in which the fast-growing co-trophic flora is more likely to increase under nutrient-rich conditions, while the oligotrophic flora increases, and the slow-growing flora (such as Chloroflexi) may decrease (Fierer et al., 2007). Chloroflexi are microorganisms that can produce energy through photosynthesis with CO_2_ as a C source, but the bacteria of this phylum have various nutritional modes, perform both aerobic and anaerobic respiration, and participate in the C and N cycles, as well as other elemental cycles, such as the S cycle (Lv et al., 2014). This is also confirmed by changes in the functional metabolism of the bacteria in this study. N application significantly reduced N and S cycling-related functions of bacterial communities (Figure 5A). The relative abundance of functional groups related to nitrate reduction decreased significantly in N treatment (Liu et al., 2020b). Environmental factors are important factors affecting the function of the microbial community. The change of gene relative abundance related to the C and N cycle is related to soil nutrient availability (Yan et al., 2020). This study found a close relationship between soil available K content and bacterial function (Figure 5B). SEM also confirmed that the loss of these functions under N application may be due to the increase of soil available K content (Figure 8). In fungi, N2 significantly increased the relative abundance of Didymosphaeriaceae, which is consistent with previous studies (Zeng et al., 2016). Other studies have found that Didymosphaeriaceae comprise saprotrophs, endophytes, or pathogens associated with various plant substrates, such as woody plant debris and rotten leaves, and herbivore feces (Ariyawansa et al., 2014; Tennakoon et al., 2016).

The change of soil microbial community structure under N application may subsequently affect the soil elements cycle and threaten the stability of the ecosystem (Tilman et al., 2006; Sun et al., 2021). The change of soil microbial community structure can also be explained by the change of soil factors. In this study, the soil bacterial community structure was mainly limited by the soil available K content (Figure 7A). This finding is consistent with previous studies (Kang et al., 2021). In alpine forest soil, the soil microbial community diversity and structure are related to the soil available K content (Wang et al., 2020). In the desert steppe region, soil available K has a significant effect on the soil bacterial community structure (Jia et al., 2019). Similar to being an essential element for plants, soil K is equally important for the soil microbial community. In this study, N2 treatment significantly promoted the soil available K content (Table 1). N application may increase the decomposition of litter and supplement the soil available K content (Song et al., 2011). At the same time, it also provided nutrition for bacteria in soil and may stimulate potassium-solubilizing bacteria, thus catalyzing the conversion of insoluble or mineral K into the soluble form in the soil. Various types of bacteria have been reported to have the ability to release K, such as Bacillus mucilaginosus and Bacillus edaphicus (Richards and Bates, 1989; Lin et al., 2002). In this study, the relative abundance of Bacillus was 1.23% on average, but N application had no significant effect on its relative abundance (Figure 3). This may be because N application may stimulate the activity of K-solubilizing bacteria, but does not increase the relative abundance.

N application significantly changed the soil bacteria and fungi community structure, and significantly changed the soil bacterial community composition and function through soil available K. At the same time, SOC also had an impact on the soil fungal community. As an explanatory variable, soil available K is related to bacterial community or functional structure, which highlights the importance of environmental factors in shaping microbial community structure and function. This study plays an important role in understanding how soil properties drive microbial community changes after N application. However, the alpine wetland ecosystem in the arid region of Central Asia is complex, and changeable environmental factors affect the soil microbial community. Therefore, long-term and more detailed research is needed to understand the driving forces and mechanisms of the soil microbial community diversity and structure in the alpine wetland ecosystem under the condition of N application.

## Conclusion

In conclusion, our results showed that short-term (1 year) N application to the swamp meadow in the arid region of Central Asia did not significantly change soil microbial community diversity but changed its composition and functional metabolism. Under the condition of N application, soil available K was the most important driving force for the change in the soil bacterial community structure. Against the background of the increasing atmospheric N deposition rate, we illustrate the importance of soil available K to the microbial community. Because the alpine wetland ecosystem in the arid region of Central Asia is vulnerable to climate change and environmental disturbance, the short-term effects of N application to swamp meadow soil are uncertain. Therefore, more attention on the long-term effects on the soil microbial community after N application is required in future research. This study can provide scientific references for climate change adaptation and management of global alpine wetlands.

## Data Availability Statement

The datasets presented in this study can be found in online repositories. The names of the repository/repositories and accession number(s) can be found below: https://www.ncbi.nlm.nih.gov/, PRJNA771427.

## Author Contributions

YH, MOC, and ZY conceived and designed the study and wrote the manuscript. YH, MOC, ZY, MEC, XZ, and HJ were responsible for performing the field and laboratory work. YH and HJ analyzed the data. All authors discussed the results, critically reviewed the manuscript, and approved its publication.

## Conflict of Interest

The authors declare that the research was conducted in the absence of any commercial or financial relationships that could be construed as a potential conflict of interest.

## Publisher’s Note

All claims expressed in this article are solely those of the authors and do not necessarily represent those of their affiliated organizations, or those of the publisher, the editors and the reviewers. Any product that may be evaluated in this article, or claim that may be made by its manufacturer, is not guaranteed or endorsed by the publisher.

## Footnotes

1
http://ccb.jhu.edu/software/FLASH/

2
http://www.arb-silva.de

3
https://unite.ut.ee/

4
http://sourceforge.net/projects/rdp-classifier

5
http://www.mothur.org/

6
http://www.loucalab.com/archive/FAPROTAX/lib/php/index.php?

7
https://github.com/UMNFuN/FUNGuild

8
https://beikolab.cs.dal.ca/software/STAMP

9
http://huttenhower.sph.harvard.edu/lefse/

## References

[B1] Allison S. D. Martiny J. B. H. (2008). Resistance, resilience, and redundancy in microbial communities. *Proc. Natl. Acad. Sci. U.S.A.* 105 11512–11519. 10.1073/pnas.0801925105 18695234 PMC2556421

[B2] Ariyawansa H. A. Camporesi E. Thambugala K. M. Mapook A. Kang J. C. Alias S. A. (2014). Confusion surrounding didymosphaeria–phylogenetic and morphological evidence suggest didymosphaeriaceae is not a distinct family. *Phytotaxa* 176 102–119. 10.11646/phytotaxa.176.1.12

[B3] Cao S. Cao G. Feng Q. Han G. Lin Y. Yuan J. (2017). Alpine wetland ecosystem carbon sink and its controls at the Qinghai Lake. *Environ. Earth Sci.* 76 1–15. 10.1007/s12665-017-6529-5

[B4] Chen M. Zhu X. Zhao C. Yu P. Maidinuer A. Jia H. (2021). Rapid microbial community evolution in initial Carex litter decomposition stages in Bayinbuluk alpine wetland during the freeze–thaw period. *Ecol. Indic.* 121:107180. 10.1016/j.ecolind.2020.107180

[B5] Christian Q. Elmar P. Pelin Y. Jan G. Timmy S. Pablo Y. (2013). The SILVA ribosomal RNA gene database project: improved data processing and web-based tools. *Nucleic Acids Res.* 41 D590–D596. 10.1093/nar/gks1219 23193283 PMC3531112

[B6] DeBruyn J. M. Radosevich M. Wommack K. E. Polson S. W. Hauser L. J. Fawaz M. N. (2014). Genome Sequence and Methylome of Soil Bacterium Gemmatirosa kalamazoonensis KBS708T, a member of the rarely cultivated gemmatimonadetes phylum. *Genome Announc.* 2:e00226-14. 10.1128/genomeA.00226-14 24699952 PMC3974934

[B7] de Vries F. T. Griffiths R. I. Bailey M. Craig H. Girlanda M. Gweon H. (2018). Soil bacterial networks are less stable under drought than fungal networks. *Nat. Commun.* 9:3033. 10.1038/s41467-018-05516-7 30072764 PMC6072794

[B8] Eichorst S. A. Trojan D. Roux S. Herbold C. Rattei T. Woebken D. (2018). Genomic insights into the Acidobacteria reveal strategies for their success in terrestrial environments. *Environ. Microbiol.* 20 1041–1063. 10.1111/1462-2920.14043 29327410 PMC5900883

[B9] Fierer N. Bradford M. A. Jackson R. B. (2007). Toward an ecological classification of soil bacteria. *Ecology* 88 1354–1364. 10.1890/05-1839 17601128

[B10] Fang D. Zhao G. Xu X. Zhang Q. Shen Q. Fang Z. (2018). Microbial community structures and functions of wastewater treatment systems in plateau and cold regions. *Bioresour. Technol.* 249 684–693. 10.1016/j.biortech.2017.10.063 29091854

[B11] Freedman Z. B. Romanowicz K. J. Upchurch R. A. Zak D. R. (2015). Differential responses of total and active soil microbial communities to long-term experimental N deposition. *Soil Biol. Biochem.* 90 275–282. 10.1016/j.soilbio.2015.08.014

[B12] Galloway J. N. Dentener F. J. Capone D. G. Boyer E. W. Howarth R. W. Seitzinger S. P. (2004). Nitrogen cycles: past, present, and future. *Biogeochemistry* 70 153–226. 10.1007/s10533-004-0370-0

[B13] Gu Y. Bai Y. Xiang Q. Yu X. Zhao K. Zhang X. (2018). Degradation shaped bacterial and archaeal communities with predictable taxa and their association patterns in Zoige wetland at Tibet plateau. *Sci. Rep.* 8 3884–3894. 10.1038/s41598-018-21874-0 29497087 PMC5832768

[B14] He J. Jiao S. Tan X. Wei H. Ma X. Nie Y. (2021). Adaptation of soil fungal community structure and assembly to long-versus short-term nitrogen addition in a tropical forest. *Front. Microbiol.* 12:2421. 10.3389/FMICB.2021.689674 34512567 PMC8424203

[B15] Huhe, Chen X. Hou F. Wu Y. Cheng Y. (2017). Bacterial and fungal community structures in loess plateau grasslands with different grazing intensities. *Front. Microbiol.* 8:606. 10.3389/fmicb.2017.00606 28439265 PMC5383705

[B16] Jia M. Huang J. Yang Y. Han G. Zhang G. (2019). Effects of simulated nitrogen deposition and precipitation manipulation on soil microorganisms in the desert steppe of northern China. *Rev. Brasil. Ciência Solo* 43:31. 10.1590/18069657rbcs20180031

[B17] Kang E. Li Y. Zhang X. Yan Z. Wu H. Li M. (2021). Soil pH and nutrients shape the vertical distribution of microbial communities in an alpine wetland. *Sci. Total Environ.* 774:145780. 10.1016/J.SCITOTENV.2021.145780

[B18] Kõljalg U. Nilsson R. H. Abarenkov K. Tedersoo L. Taylor A. F. S. Bahram M. (2013). Towards a unified paradigm for sequence-based identification of fungi. *Mol. Ecol.* 22 5271–5277. 10.1111/mec.12481 24112409

[B19] Leps J. Smilauer P. (2003). *Multivariate Analysis of Ecological Data Using CANOCO.* Cambridge: Cambridge University Press.

[B20] Li J. Sang C. Yang J. Qu L. Xia Z. Sun H. (2021). Stoichiometric imbalance and microbial community regulate microbial elements use efficiencies under nitrogen addition. *Soil Biol. Biochem.* 156:108207. 10.1016/J.SOILBIO.2021.108207

[B21] Li J. Yang C. Zhou H. Shao X. (2020). Responses of plant diversity and soil microorganism diversity to water and nitrogen additions in the Qinghai-Tibetan Plateau. *Glob. Ecol. Conserv.* 22:e01003. 10.1016/j.gecco.2020.e01003

[B22] Li K. Gong Y. Song W. He G. Hu Y. Tian C. (2012). Responses of CH4, CO2 and N2O fluxes to increasing nitrogen deposition in alpine grassland of the Tianshan mountains. *Chemosphere* 88 140–143. 10.1016/j.chemosphere.2012.02.077 22445955

[B23] Li Y. Wang S. Jiang L. Zhang L. Cui S. Meng F. (2016). Changes of soil microbial community under different degraded gradients of alpine meadow. *Agric. Ecosyst. Environ.* 222 213–222. 10.1016/j.agee.2016.02.020

[B24] Louca S. Parfrey L. W. Doebeli M. (2016). Decoupling function and taxonomy in the global ocean microbiome. *Science* 353 1272–1277. 10.1126/science.aaf4507 27634532

[B25] Lin Q. Rao Z. Sun Y. Yao J. Xing L. (2002). Identification of a silicate-dissolving bacterium and its effect on tomato. *Sci. Agric. Sin.* 35 59–62. 10.1006/jfls.2001.0409

[B26] Liu X. Zhang S. (2019). Nitrogen addition shapes soil enzyme activity patterns by changing pH rather than the composition of the plant and microbial communities in an alpine meadow soil. *Plant Soil* 440 11–24. 10.1007/s11104-019-04054-5

[B27] Liu W. Jiang L. Yang S. Wang Z. Tian R. Peng Z. (2020a). Critical transition of soil bacterial diversity and composition triggered by nitrogen enrichment. *Ecology* 101:e03053. 10.1002/ecy.3053 32242918

[B28] Liu W. Ling N. Guo J. Yang R. Zhu C. Shen Q. (2020b). Legacy effects of 8-year nitrogen inputs on bacterial assemblage in wheat rhizosphere. *Biol. Fertil. Soils* 56 583–596. 10.1007/s00374-020-01435-2

[B29] LiuSui Y. Zhu X. Li D. Yan C. Sun T. Jia H. (2019). Soil aggregate and intra-aggregate carbon fractions associated with vegetation succession in an alpine wetland of Northwest China. *Catena* 181 104–107. 10.1016/j.catena.2019.104107

[B30] Lv X. Yu J. Fu Y. Ma B. Qu F. Ning K. (2014). A Meta-Analysis of the bacterial and archaeal diversity observed in wetland soils. *Sci. World J.* 2014:437684. 10.1155/2014/437684 24982954 PMC4058131

[B31] Li B. Roley S. S. Duncan D. S. Guo J. Quensen J. F. Yu H. (2021). Long-term excess nitrogen fertilizer increases sensitivity of soil microbial community to seasonal change revealed by ecological network and metagenome analyses. *Soil Biol. Biochem.* 160:108349. 10.1016/j.soilbio.2021.108349

[B32] McHugh T. A. Morrissey E. M. Mueller R. C. Gallegos-Graves L. V. Kuske C. R. Reed S. C. (2017). Bacterial, fungal, and plant communities exhibit no biomass or compositional response to two years of simulated nitrogen deposition in a semiarid grassland. *Environ. Microbiol.* 19 1600–1611. 10.1111/1462-2920.13678 28120480

[B33] Moore J. A. M. Anthony M. A. Pec G. J. Trocha L. K. Trzebny A. Geyer K. M. (2020). Fungal community structure and function shifts with atmospheric nitrogen deposition. *Glob. Chang. Biol.* 27 1349–1364. 10.1111/GCB.15444 33159820

[B34] Peter H. (2007). Environmental science: nitrogen impacts on forest carbon. *Nature* 447 781–782. 10.1038/447781a 17568730

[B35] Richards J. E. Bates T. E. (1989). Studies on the potassium-supplyingcapacities of southern Ontario soils. III. Measurement of available K. *Can. J. Soil Sci.* 69 597–610. 10.4141/cjss89-060

[B36] Ramirez K. S. Lauber C. L. Knight R. Bradford M. A. Fierer N. (2010). Consistent effects of nitrogen fertilization on soil bacterial communities in contrasting systems. *Ecology* 91 3463–3470. 10.1890/10-0426.1 21302816

[B37] Raza S. Na M. Wang P. Ju X. Chen Z. Zhou J. (2020). Dramatic loss of inorganic carbon by nitrogen-induced soil acidification in Chinese croplands. *Glob. Chang. Biol.* 26 3738–3751. 10.1111/gcb.15101 32239592

[B38] Shade A. Peter H. Allison S. D. Baho D. Berga M. Buergmann H. (2012). Fundamentals of microbial community resistance and resilience. *Front. Microbiol.* 3:417. 10.3389/fmicb.2012.00417 23267351 PMC3525951

[B39] Segata N. Izard J. Waldron L. Gevers D. Miropolsky L. Garrett W. (2011). Metagenomic biomarker discovery and explanation. *Genome Biol.* 12:R60. 10.1186/gb-2011-12-6-r60 21702898 PMC3218848

[B40] Sessitsch A. Weilharter A. Gerzabek M. H. Kirchmann H. Kandeler E. (2001). Microbial population structures in soil particle size fractions of a long-term fertilizer field experiment. *Appl. Environ. Microbiol.* 67 4215–4224. 10.1128/AEM.67.9.4215-4224.2001 11526026 PMC93150

[B41] Sun R. Wang F. Hu C. Liu B. (2021). Metagenomics reveals taxon-specific responses of the nitrogen-cycling microbial community to long-term nitrogen fertilization. *Soil Biol. Biochem.* 156:108214. 10.1016/j.soilbio.2021.108214

[B42] Shao K. Bai C. Cai J. Hu Y. Gong Y. Chao J. (2019). Illumina sequencing revealed soil microbial communities in a Chinese alpine grassland. *Geomicrobiol. J.* 36 204–211. 10.1080/01490451.2018.1534902

[B43] Shao K. Gao G. (2018). Soil microbial communities of three grassland ecosystems in the Bayinbuluke, China. *Can. J. Microbiol.* 64 209–213. 10.1139/cjm-2017-0585 29206480

[B44] Shi Y. Sheng L. Wang Z. Zhang X. He N. Yu Q. (2016). Responses of soil enzyme activity and microbial community compositions to nitrogen addition in bulk and microaggregate soil in the temperate steppe of Inner Mongolia. *Eurasian Soil Sci.* 49 1149–1160. 10.1134/S1064229316100124

[B45] Song C. Liu D. Yang G. Song Y. Mao R. (2011). Effect of nitrogen addition on decomposition of calamagrostis angustifolia litters from freshwater marshes of northeast China. *Ecol. Eng.* 37 1578–1582. 10.1016/j.ecoleng.2011.03.036

[B46] Tilman D. Reich P. B. Knops J. M. H. (2006). Biodiversity and ecosystem stability in a decade-long grassland experiment. *Nature* 441 629–632. 10.1038/nature04742 16738658

[B47] Tennakoon D. S. Hyde K. D. Wanasinghe D. N. Bahkali A. H. Phookamsak R. (2016). Taxonomy and phylogenetic appraisal of *Montagnula jonesii* sp. nov. (Didymosphaeriaceae. Pleosporales). *Mycosphere* 7 1346–1356. 10.5943/mycosphere/7/9/8

[B48] Vitousek P. M. Porder S. Houlton B. Z. Chadwick O. A. (2010). Terrestrial phosphorus limitation: mechanisms, implications, and nitrogen–phosphorus interactions. *Ecol. Applic.* 20 5–15. 10.1890/08-0127.1 20349827

[B49] Wang C. Qu L. Yang L. Liu D. Morrissey E. Miao R. (2021). Large-scale importance of microbial carbon use efficiency and necromass to soil organic carbon. *Glob. Change Biol.* 27 2039–2048. 10.1111/GCB.15550 33559308

[B50] Wang H. Liu S. Zhang X. Mao Q. Li X. You Y. (2018). Nitrogen addition reduces soil bacterial richness, while phosphorus addition alters community composition in an old-growth N-rich tropical forest in southern China. *Soil Biol. Biochem.* 127 22–30. 10.1016/j.soilbio.2018.08.022

[B51] Wang C. Liu D. Bai E. (2018). Decreasing soil microbial diversity is associated with decreasing microbial biomass under nitrogen addition. *Soil Biol. Biochem.* 120 126–133. 10.1016/j.soilbio.2018.02.003

[B52] Wang X. Zhang Z. Yu Z. Shen G. Cheng H. Tao S. (2020). Composition and diversity of soil microbial communities in the alpine wetland and alpine forest ecosystems on the Tibetan Plateau. *Sci. Total Environ.* 747 141358. 10.1016/j.scitotenv.2020.141358 32771793

[B53] Wu J. Liu W. Zhang W. Shao Y. Duan H. Chen B. (2019). Long-term nitrogen addition changes soil microbial community and litter decomposition rate in a subtropical forest. *Appl. Soil Ecol.* 142 43–51. 10.1016/j.apsoil.2019.05.014

[B54] Xie F. Ma A. Zhou H. Liang Y. Yin J. Ma K. (2020). Revealing fungal communities in alpine wetlands through species diversity, functional diversity and ecological network diversity. *Microorganisms* 8:632. 10.3390/microorganisms8050632 32349397 PMC7284966

[B55] Xiong J. Peng F. Sun H. Xue X. Chu H. (2014). Divergent responses of soil fungi functional groups to short-term warming. *Microb. Ecol.* 68 708–715. 10.1007/s00248-014-0385-6 24553914

[B56] Xiao Y. Li C. Yang Y. Peng Y. Yang Y. Zhou G. (2020). Soil fungal community composition, not assembly process, was altered by nitrogen addition and precipitation changes at an alpine steppe. *Front. Microbiol.* 11:579072. 10.3389/fmicb.2020.579072 33178161 PMC7597393

[B57] Yuan X. Knelman J. E. Gasarch E. Wang D. Nemergut D. R. Seastedt T. R. (2016). Plant community and soil chemistry responses to long-term nitrogen inputs drive changes in alpine bacterial communities. *Ecology* 97 1543–1554. 10.1890/15-1160.1 27459784

[B58] Yan B. S. Sun L. P. Li J. J. Liang C. Q. Wei F. R. Xue S. (2020). Change in composition and potential functional genes of soil bacterial and fungal communities with secondary succession in Quercus liaotwigensis forests of the Loess Plateau, western China. *Geoderma* 364:114199. 10.1016/j.geoderma.2020.114199

[B59] Yang Y. Wu P. (2020). Soil bacterial community varies but fungal community stabilizes along five vertical climate zones. *Catena* 195:104841. 10.1016/j.catena.2020.104841

[B60] Zeng J. Liu X. Song L. Lin X. Zhang H. Shen C. (2016). Nitrogen fertilization directly affects soil bacterial diversity and indirectly affects bacterial community composition. *Soil Biol. Biochem.* 92 41–49. 10.1016/j.soilbio.2015.09.018

[B61] Zhang H. Wang L. Liu H. Zhao J. Li G. Wang H. (2018). Nitrogen deposition combined with elevated precipitation is conducive to maintaining the stability of the soil fungal diversity on the Stipa baicalensis steppe. *Soil Biol. Biochem.* 117 135–138. 10.1016/j.soilbio.2017.11.004

[B62] Zhou Z. Wang C. Luo Y. (2020). Meta-analysis of the impacts of global change factors on soil microbial diversity and functionality. *Nat. Commun.* 11:3072. 10.1038/s41467-020-16881-7 32555185 PMC7300008

[B63] Zhou H. Zhang D. Jiang Z. Sun P. Xiao H. Wu Y. (2019). Changes in the soil microbial communities of alpine steppe at Qinghai-Tibetan Plateau under different degradation levels. *Sci. Total Environ.* 651 2281–2291. 10.1016/j.scitotenv.2018.09.336 30326458

[B64] Zhou Z. Wang C. Zheng M. Luo Y. (2017). Patterns and mechanisms of responses by soil microbial communities to nitrogen addition. *Soil Biol. Biochem.* 115 433–441. 10.1016/j.soilbio.2017.09.015

[B65] Zhu J. Chen Z. Wang Q. Xu L. He N. Jia Y. (2019). Potential transition in the effects of atmospheric nitrogen deposition in China. *Environ. Pollut.* 258:113739. 10.1016/j.envpol.2019.113739 31874437

[B66] Zhu Y. Peng J. Wei Z. Shen Q. Zhang F. (2020). Linking the soil microbiome to soil health. *Sci. Sin. Vitae* 51 1–11. 10.1360/SSV-2020-0320

